# *MTHFR* gene polymorphisms and susceptibility to myocardial infarction: Evidence from meta-analysis and trial sequential analysis

**DOI:** 10.1016/j.ijcha.2023.101293

**Published:** 2023-11-22

**Authors:** Amir Samii, Saeed Aslani, Danyal Imani, Bahman Razi, Seyedeh Samaneh Tabaee, Tannaz Jamialahmadi, Amirhossein Sahebkar

**Affiliations:** a Department of Hematology and Blood Transfusion, School of Allied Medical Science, Iran University of Medical Sciences, Tehran, Iran; b Department of Immunology, School of Medicine, Tehran University of Medical Sciences, Tehran, Iran; c Department of Immunology, School of Public Health, Tehran University of Medical Sciences, Tehran, Iran; d Department of Hematology and Blood Transfusion, Faculty of Medical Sciences, Tarbiat Modares University (TMU), Tehran, Iran; e Noncommunicable Disease Research Center, Neyshabur University of Medical Sciences, Neyshabur, Iran; f Faculty of Medicine, Neyshabur University of Medical Sciences, Neyshabur, Iran; g International UNESCO Center for Health-Related Basic Sciences and Human Nutrition, Mashhad University of Medical Sciences, Mashhad, Iran; h Biotechnology Research Center, Pharmaceutical Technology Institute, Mashhad University of Medical Sciences, Mashhad, Iran; i Applied Biomedical Research Center, Mashhad University of Medical Sciences, Mashhad, Iran

**Keywords:** Myocardial infarction, Methylenetetrahydrofolate reductase, MI, Meta-analysis, Genetic polymorphism, Heterogeneity

## Abstract

**Background:**

This meta-analysis aimed to provide a comprehensive assessment of the association between Methylenetetrahydrofolate reductase (*MTHFR*) gene polymorphisms, specifically C677T and A1298C, and the susceptibility to myocardial infarction (MI).

**Methods:**

A systematic literature search was conducted in MEDLINE, Web of Science, and Scopus until April 2023 to identify studies investigating the relationship between *MTHFR* gene polymorphisms (C677T and A1298C) and the risk of MI.

**Results:**

The analysis included 66 studies involving 16,860 cases and 20,403 controls for the C677T polymorphism and 18 studies comprising 3162 cases and 3632 controls for the A1298C polymorphism. Significant associations were observed between the C677T polymorphism and MI risk in various genetic models: dominant (OR = 1.16, 95 % CI = 1.06–1.28, *P* = 0.008), recessive (OR = 1.20, 95 % CI = 1.12–1.28, *P* < 0.001), allelic (OR = 1.13, 95 % CI = 1.06–1.21, *P* < 0.001), TT *vs*. CC (OR = 1.19, 95 % CI = 1.05–1.36, *P* < 0.001), and CT *vs*. CC (OR = 1.11, 95 % CI = 1.02–1.21, *P* = 0.01). Furthermore, an overall analysis indicated a marginally significant association between the A1298C polymorphism and MI risk in the recessive model (OR = 1.27, 95 % CI = 1.06–1.51, *P* = 0.008), allelic model (OR = 1.18, 95 % CI = 1.01–1.39, *P* = 0.03), and CC *vs*. AA model (OR = 1.22, 95 % CI = 1.01–1.47, *P* = 0.04). Meta-regression analysis revealed that none of the potential factors contributed to the observed heterogeneity.

**Conclusions:**

This meta-analysis revealed an association between *MTHFR* gene C677T and A1298C polymorphisms and the risk of MI.

## 1 Introduction

Myocardial infarction (MI) is a prevalent cardiovascular disease (CVD) worldwide and remains a significant public health concern due to its leading role in mortality in many countries [1], [2]. MI occurs when the blood supply to the heart is disrupted, primarily caused by the formation of a thrombus at the site of plaque erosion within the coronary arteries, resulting in the death of heart muscle cells [3]. Recent data from the National Health and Nutrition Examination Survey (NHANES) showed a prevalence of 3.6 percent for MI in the United States, with rates of 4.7 percent for males and 2.6 percent for females [4]. Although the exact cause of MI is still unknown, there is growing evidence that a complex interplay between genetic predisposition and environmental factors contributes to susceptibility to this disorder [5], [6], [7]. While common risk factors such as obesity, hypertension, diabetes, dyslipidemia, and smoking account for a portion of the cardiovascular risk, there is also a residual component that is strongly influenced by genetic factors [8]. Extensive research has focused on identifying genetic variations and polymorphisms that play a role in crucial processes, including coagulation, blood pressure regulation, and metabolic pathways (such as carbohydrate, lipid, and homocysteine metabolism), which may increase an individual's susceptibility to MI [9], [10]. However, the exact mechanisms underlying these associations have yet to be fully understood.

Methylenetetrahydrofolate reductase (MTHFR) is a crucial enzyme involved in the folate metabolic pathway, which plays a vital role in methyl group transportation [11]. It catalyzes the conversion of 5,10-methylenetetrahydrofolate (5,10-MTHF) to 5-methyltetrahydrofolate (5-MTHF), an essential step in the synthesis of methionine from homocysteine [12]. This conversion produces 5-MTHF, an active form of folate required for the methylation of homocysteine to methionine [12]. Genetic polymorphisms that reduce MTHFR enzyme activity lead to the accumulation of homocysteine [13]. Interestingly, elevated levels of plasma homocysteine have been linked to the pathophysiology of various disorders, including MI [14], [15], [16]. Two common single nucleotide polymorphisms (SNPs) have been identified within the MTHFR gene: C677T (*rs1801133*) and A1298C (*rs1801131*). These SNPs influence MTHFR enzyme activity and have been associated with the risk of CVD [17]. The C677T polymorphism, resulting in an Alanine-to-Valine substitution, and the A1298C polymorphism, resulting in a Glutamate-to-Alanine substitution, may decrease MTHFR enzyme activity, leading to increased serum homocysteine levels [18], [19]. Elevated homocysteine levels are associated with an increased risk of atherosclerosis, a condition characterized by the buildup of plaques in the arteries. Homocysteine is believed to contribute to atherosclerosis by damaging the inner lining of blood vessels and promoting inflammation, which can lead to the formation of atherosclerotic plaques. These plaques can eventually rupture, causing the formation of blood clots that can block coronary arteries and result in MI [20].

Several studies have examined the association between the *MTHFR* gene C677T and A1298C SNPs and the risk of MI [21], [22]. However, the findings have been inconclusive and inconsistent due to factors such as small sample sizes, ethnic diversity in the populations studied, variations in linkage disequilibrium (LD) between genotypes, inadequate statistical power, and challenges in defining the disease phenotype. Therefore, to obtain a more accurate understanding of the association between the *MTHFR* gene C677T and A1298C polymorphisms and the risk of MI, a comprehensive meta-analysis was conducted, incorporating all eligible case-control studies that had been published.

## 2 Methods

This study was designed and conducted based on the PRISMA statement [23]. The study does not contain any experiment on animals or human. Therefore, there is no need for ethical approval.

### 2.1 Search strategy

The search procedure involved a thorough and systematic exploration of pertinent literature across multiple databases, including PubMed, Web of Science, and Scopus to retrieve all potential literatures evaluating C677T and/or A1298C polymorphisms and susceptibility to MI. Key words and Medical Subject Headings (Mesh) terms were as: (“Methylenetetrahydrofolate Reductase” OR “MTHFR” OR “C667T” OR “A1298C”) AND (“SNP” OR “single nucleotide polymorphism” OR “polymorphisms” OR “variation” OR “mutation”) AND (“Myocardial Infarction” OR “Acute coronary syndrome” OR “ischemic heart disease” OR “coronary artery disease”) (Supplementary file). The primary search was conducted without broad restriction filters to focus on human studies and publications in the English language that encompassed publications from their inception until April 2023. Moreover, potential missing publications were detected by cross-checking the references of review articles.

### 2.2 Inclusion and exclusion criteria

To qualify for consideration, it is necessary to fulfill the following set of criteria: 1) publications with case-control design; 2) All publications evaluated the association between *MTHFR* gene C677T and/or A1298C polymorphisms and susceptibility to MI; 3) Any publications that provide the necessary information to derive or compute risk estimates alongside their corresponding 95 % confidence intervals are included. The studies were excluded if were: books, letters, case reports, duplicate publications, review, study on animal, and studies with insufficient data. Upon initial search, to ensure the highest level of quality and relevance, we systematically assessed each study's title, abstract, and keywords. Irrelevant and duplicative studies were promptly excluded at this stage. Following this initial screening, the remaining studies underwent a thorough full-text review, during which our rigorous inclusion and exclusion criteria were meticulously applied to ascertain that only studies of the utmost relevance and scientific merit were incorporated. The application of these criteria yelled 66 publications for C677T SNP and 18 publications for A1298C SNP.

### 2.3 Data extraction and quality assessment

The data extraction process involved two independent investigators (DI and BR) who acquired full-text of eligible publications. Cohen’s kappa coefficient was applied to examine the differences between the two authors in the selection of studies and in the evaluation of the quality of evidence for the studies. In case of no agreement on selection of a publication, we achieved consensus through comprehensive discussions in the presence of third researcher, thereby establishing a unified approach to study selection. This collaborative effort ensures the methodological rigor and reliability of our meta-analysis, strengthening the validity of the results presented in this study. The data that was taken out includes: author’s last name, the country study, sample size, year of publication, ethnicity of subjects, age, method of genotyping, and genotype and allele distribution in both the case as well as control group. The methodological quality of included studies was assessed using the Newcastle-Ottawa Scale (NOS) [24]. Moreover, three major items were: comparability, ascertainment of outcome, and selection. This scale ranges between one to nine stare and publications with scores 0–3, 4–6, or 7–9 was of low, moderate, or high-quality, respectively.

### 2.4 Trial sequential analysis

Trial sequential analysis (TSA) is a statistical approach that combines the calculation of information size (total sample sizes from all included publications) in order to reduce both type I (alpha) and type II (beta) errors in meta-analysis, while maintaining a threshold for statistical significance. The required information size (RIS) was determined with a 5 % level of type I error, 80 % statistical power, a relative risk reduction (RRR) of 20 %, and a two-sided boundary type. If the cumulative Z‐curve crosses the TSA monitoring boundary or exceeds the RIS, which indicates sufficient evidence, further studies are not needed. The reverse condition necessitates continuous trials.

### 2.5 Statistical analysis

In the control group, Pearson's chi square test was applied to assess the deviation from Hardy–Weinberg equilibrium (HWE) for genotype frequency (*P* < 0.05 was considered significant). The five models of *MTHFR* gene C677T and A1298C polymorphisms included dominant, allelic, recessive, heterozygote, and homozygote models. The extent of association between models of each *MTHFR* gene polymorphisms and MI risk was evaluated through pooled odds ratio (ORs) and their 95 % confidence intervals (CIs). Cochrane's Q test and the I^2^ test were conducted to find between study heterogeneity. The fixed-effected model (FEM) was applied if *P*_Q-statistic_ > 0.10 or I^2^ was < 50 %; any other way, the random-effected model (REM) was used [25], [26]. To evaluate the predefined sources of heterogeneity among the studies, various approaches were employed. Subgroup analysis was conducted based on ethnicity, and meta-regression analysis was performed according to year of publication and subjects’ ethnicity. Sensitivity analysis was utilized to determine the impact of individual studies on the overall effect size. To assess publication bias, Begg's and Egger's tests, and a visual inspection of the funnel plot were conducted [27], [28]. The statistical analysis was performed using both STATA statistical software and SPSS.

## 3 Results

### 3.1 Study characteristic and search results

**Fig. 1:**
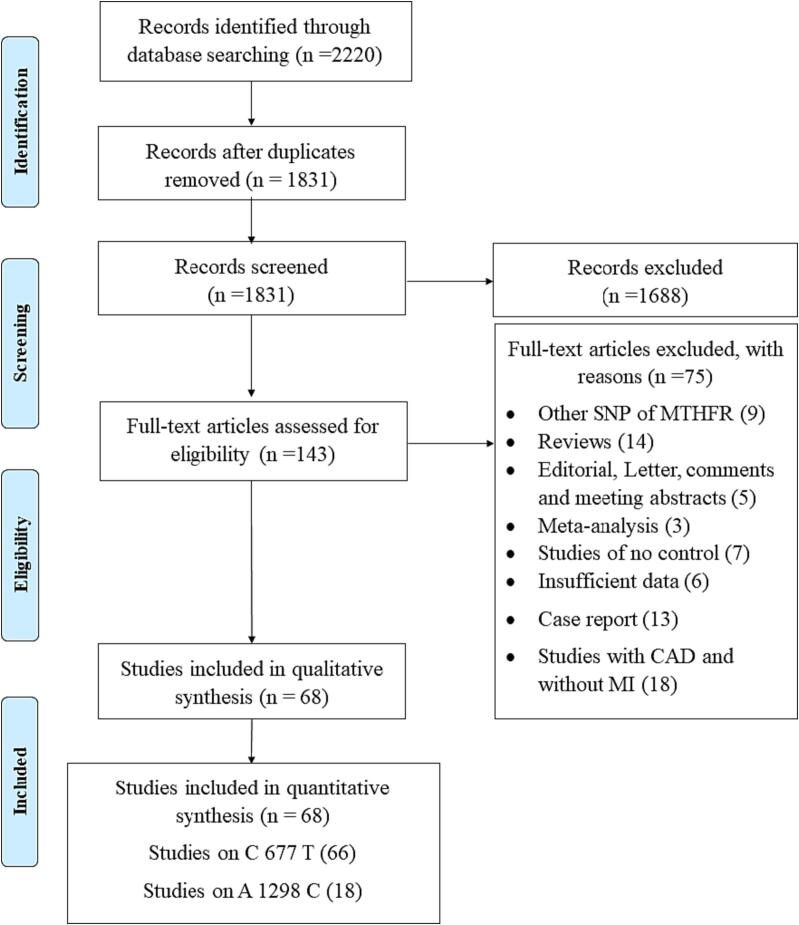
Flow diagram of study selection process.

Briefly, the initial search through three major databases yielded 2220 publications. After removing the duplicates (n = 389), 1831 manuscripts were screened based on the titles and abstracts, from which 1688 were excluded. The remaining 143 manuscripts were screened and assessed based on the full text for eligibility. Eventually, 66 studies for C677T and 18 studies for A1298C qualified for quantitative analysis [21], [29], [30], [31], [32], [33], [34], [35], [36], [37], [38], [39], [40], [41], [42], [43], [44], [45], [46], [47], [48], [49], [50], [51], [52], [53], [54], [55], [56], [57], [58], [59], [60], [61], [62], [63], [64], [65], [66], [67], [68], [69], [70], [71], [72], [73], [74], [75], [76], [77], [78], [79], [80], [81], [82], [83], [84], [85], [86], [87], [88], [89], [90], [91], [92], [93], [94], [95], [96]. The reasons for exclusion at the level of full text assessment are shown in Fig. 1. After cross-checking the references of all eligible publications, no additional studies were discovered. Of 68 eligible studies, 16 of them evaluated C677T and A1298C SNPs simultaneously and the other 52 studies involved C677T SNP. The included literatures were published between 1996 until 2022 and all studies reached a good NOS ranging 5 to 8. The result of Cohen’s kappa assessment was 0.964, suggesting an almost perfect agreement between the two authors. Table 1, Table 2 summarize the main original data for both SNPs.

**Table 1:** Characteristics of studies included in meta-analysis.

**Study author**	**Year**	**Country**	**Ethnicity**	**Sex**	**Total cases/controls**	**Quality** **score**	
**MTHFR C677T**							
Adams et al.	1996	England	European	Male/Female	310 / 222	7	
Schmitz *et al.*	1996	USA	Mixed	Male/Female	190 / 188	6	
Ma *et al.*	1996	USA	Mixed	Male	293 / 290	7	
Brugada *et al.*	1997	USA	Mixed	Male/Female	79 / 155	5	
Anderson *et al.*	1997	USA	Mixed	Male/Female	200 / 168	6	
Schwartz *et al.*	1997	USA	Mixed	Female	79 / 386	6	
Girelli *et al.*	1998	Italy	European	Male/Female	183 / 137	6	
Abbate *et al.*	1998	Italy	European	Male/Female	38 / 106	5	
Ferrer-Antunes *et al.*	1998	Portugal	European	Male/Female	127 / 127	5	
Ardissino *et al.*	1999	Italy	European	Male/Female	200 / 200	6	
Gardemann *et al.*	1999	Germany	European	Male	1152 / 185	7	
Fernandez-Arcas *et al.*	1999	Spain	European	Male/Female	272 / 472	7	
Virgos *et al.*	2000	Spain	European	Male	72 / 72	5	
Nakai *et al.*	2000	Japan	Asian	Male	230 / 198	6	
Thogersen *et al.*	2001	Sweden	European	Male/Female	69 / 129	5	
Zhang *et al.*	2001	China	Asian	Male/Female	73 / 100	5	
Dilley *et al.*	2001	USA	Mixed	Male/Female	110 / 185	5	
Gulec *et al.*	2001	Turkey	European	Male	96 / 100	5	
Mukherjee *et al.*	2002	India	Asian	Male/Female	83 / 205	5	
Ranjith *et al.*	2003	South Africa	African	Male/Female	195 / 300	6	
Italian Study Group	2003	Italy	European	Male/Female	1210 / 1210	8	
Tanis *et al.*	2004	Netherlands	European	Female	181 / 601	6	
El-Sammak *et al.*	2004	Egypt	African	Male	50 / 50	5	
Shioji *et al.*	2004	Japan	Asian	Male/Female	548 / 1857	5	
Khare *et al.*	2004	India	Asian	Male/Female	120 / 100	7	
Angeline *et al.*	2004	India	Asian	NR	52 / 20	6	
Helfenstein *et al.*	2005	Brazil	Mixed	Male/Female	47 / 56	6	
Iqbal *et al.*	2005	Pakistan	Asian	Male/Female	397 / 225	7	
Karakuş *et al.*	2005	Turkey	European	Male/Female	63 / 81	5	
Yamada *et al.*	2006	Japan	Asian	Male/Female	1192 / 2291	8	
Angeline *et al.*	2007	India	Asian	Male/Female	100 / 100	5	
Celik *et al.*	2008	Turkey	European	Male/Female	129 / 107	6	
Beletic *et al.*	2009	Serbia	European	Male/Female	86 / 35	5	
Koubaa *et al.*	2010	Tunisia	African	Male/Female	59 / 118	6	
Isordia-Salas *et al.*	2010	Mexico	Mixed	Male/Female	167 / 167	6	
Dayakar *et al.*	2011	India	Asian	Male/Female	152 / 167	6	
Balogh *et al.*	2012	Hungary	European	Male/Female	34 / 302	5	
Eftychiou *et al.*	2012	Greece	European	Male	63 / 54	5	
Dogra *et al.*	2012	India	Asian	Male/Female	184 / 350	6	
Tomaiuolo *et al.*	2012	Italy	European	Male/Female	1626 / 909	8	
Söderström *et al.*	2013	Sweden	European	Male/Female	514 / 971	8	
Sakowicz *et al.*	2013	Poland	European	Male/Female	272 / 141	6	
slama *et al.*	2013	Tunisia	African	Male/Female	100 / 200	6	
Mehlig *et al.*	2013	Sweden	European	Male/Female	856 / 1129	8	
Mehlig *et al.*	2013	Sweden	European	Male/Female	294 / 624	7	
Shaker *et al.*	2014	Egypt	African	Male/Female	60 / 60	5	
Ezzat *et al.*	2014	Egypt	African	Male/Female	30 / 15	5	
Al-Gazally *et al.*	2015	Iraq	Asian	Male/Female	30 / 30	5	
Alkhiary *et al.*	2015	Egypt	African	Male/Female	31 / 20	5	
Senol *et al.*	2015	Turkey	European	NR	70 / 70	5	
Hmimech *et al.*	2016	Morocco	African	NR	100 / 182	6	
Iqbal *et al.*	2016	Pakistan	Asian	Male/Female	143 / 154	6	
Zhang *et al.*	2016	China	Asian	Male/Female	304 / 551	7	
Hashad *et al.*	2017	Egypt	African	Male/Female	100 / 100	5	
Garcia-Gonzalez *et al.*	2018	Mexico	Mixed	Male/Female	79 / 101	5	
Eissa *et al.*	2019	Iraq	Asian	Male/Female	75 / 75	5	
Hou *et al.*	2019	china	Asian	NR	860 / 1097	8	
Zhang *et al.*	2019	China	Asian	Male/Female	875/ 956	8	
Long *et al.*	2019	China	Asian	Male/Female	406/231	7	
Raina *et al.*	2020	India	Asian	Male/Female	400/400	7	
Ameen *et al.*	2020	Iraq	Asian	Male/Female	102/77	5	
Bouzidi *et al.*	2020	Tunisia	African	Male/Female	310/207	6	
Shivkar *et al.*	2021	India	Asian	Male/Female	45/45	5	
Golestani *et al.*	2022	Iran	Asian	Male/Female	150/150	6	
Sugijo *et al.*	2022	Indonesia	Asian	Male/Female	30/30	5	
Timizheva *et al.*	2022	Russia	European	Male/Female	113/62	5	
**MTHFR A1298C**							
Ranjith *et al.*	2003	South Africa	African	Male/Female	195 / 300	7	
Angeline *et al.*	2004	India	Asian	NR	52 / 20	5	
Dayakar *et al.*	2011	India	Asian	Male/Female	152 / 167	6	
Eftychiou *et al.*	2012	Greece	European	Male	63 / 54	5	
slama *et al.*	2013	Tunisia	African	Male/Female	100 / 200	6	
Soderstrom *et al.*	2013	Sweden	European	Male/Female	517 / 970	8	
Nasiri *et al.*	2014	Iran	Asian	Male/Female	54 / 54	5	
Alkhiary *et al.*	2015	Egypt	African	Male/Female	31 / 20	5	
Senol *et al.*	2015	Turkey	European	Male/Female	70 / 70	5	
Iqbal *et al.*	2016	Pakistan	Asian	Male/Female	143 / 154	6	
Garcia-Gonzalez *et al.*	2018	Mexico	Mixed	Male/Female	79 / 101	5	
Eissa *et al.*	2019	Iraq	Asian	Male/Female	75 / 75	5	
Zhang *et al.*	2019	China	Asian	Male/Female	875/956	8	
Long *et al.*	2019	China	Asian	Male/Female	406/231	7	
Amani *et al.*	2020	Iran	Asian	Male/Female	90/76	5	
Shivkar *et al.*	2021	India	Asian	Male/Female	45/45	5	
Ameen *et al.*	2021	Iraq	Asian	Male/Female	102/77	5	
Timizheva *et al.*	2022	Russia	European	Male/Female	113/62	5

**Table 2:** Distribution of genotype and allele among MI patients and controls.

**Study author**	**MI cases**					**Healthy control**					**P-HWE**	**MAF**
	**CC**	**CT**	**TT**	**C**	**T**	**CC**	**CT**	**TT**	**C**	**T**		
Adams *et al.*	133	145	32	411	209	96	97	29	289	155	0/566	0/35
Schmitz *et al.*	95	66	29	256	124	71	90	27	232	144	0/859	0/38
Ma *et al.*	136	124	33	396	190	135	116	39	386	194	0/084	0/33
Brugada *et al.*	41	32	6	114	44	70	73	12	213	97	0/235	0/31
Anderson *et al.*	90	87	23	267	133	73	73	22	219	117	0/579	0/35
Schwartz *et al.*	28	34	7	90	48	154	141	43	449	227	0/233	0/33
Girelli *et al.*	60	93	30	213	153	42	70	25	154	120	0/658	0/43
abbate *et al.*	11	17	10	39	37	26	48	32	100	112	0/347	0/53
Ferrer-Antunes *et al.*	54	59	14	167	87	71	51	5	193	61	0/258	0/24
Ardissino *et al.*	68	97	35	233	167	60	102	38	222	178	0/646	0/44
Gardemann *et al.*	527	515	110	1569	735	88	82	15	258	112	0/496	0/3
Fernandez-Arcas *et al.*	105	107	60	317	227	166	216	90	548	396	0/189	0/42
Virgos *et al.*	34	33	5	101	43	27	31	14	85	59	0/351	0/41
Nakai *et al.*	93	95	42	281	179	81	96	21	258	138	0/34	0/35
Thogersen *et al.*	32	32	5	96	42	71	51	7	193	65	0/578	0/25
Zhang *et al.*	32	33	8	97	49	37	47	16	121	79	0/867	0/39
Dilley *et al.*	91	17	2	199	21	153	28	4	334	36	0/059	0/1
Gulec *et al.*	42	39	15	123	69	60	35	5	155	45	0/971	0/22
Mukherjee *et al.*	53	30	0	136	30	137	63	5	337	73	0/474	0/18
Ranjith *et al.*	166	29	0	361	29	238	58	4	534	66	0/827	0/11
Italian Study Group	371	547	292	1289	1131	363	617	230	1343	1077	0/261	0/44
Tanis *et al.*	78	81	22	237	125	280	262	59	822	380	0/84	0/32
El-Sammak *et al.*	22	22	6	66	34	22	24	4	68	32	0/466	0/32
Shioji *et al.*	193	250	88	636	426	663	878	305	2204	1488	0/618	0/4
Khare *et al.*	100	19	1	219	21	86	14	0	186	14	0/451	0/07
Angeline *et al.*	41	10	1	92	12	17	3	0	37	3	0/716	0/07
Helfenstein *et al.*	21	21	5	63	31	26	24	6	76	36	0/895	0/32
Iqbal *et al.*	279	110	8	668	126	161	57	7	379	71	0/482	0/16
Karakuş *et al.*	24	33	6	81	45	38	36	7	112	50	0/709	0/31
Yamada *et al.*	375	570	247	1320	1064	804	1134	353	2742	1840	0/152	0/4
Angeline *et al.*	81	18	1	180	20	84	16	0	184	16	0/384	0/08
Celik *et al.*	72	47	10	191	67	66	34	7	166	48	0/369	0/22
Beletic *et al.*	43	36	7	122	50	16	13	6	45	25	0/258	0/36
Koubaa *et al.*	29	17	13	75	43	73	36	9	182	54	0/14	0/45
Isordia-Salas *et al.*	38	75	54	151	183	42	78	47	162	172	0/4	0/51
Dayakar *et al.*	115	35	2	265	39	159	8	0	326	8	0/751	0/02
Balogh *et al.*	15	17	2	47	21	137	133	32	407	197	0/973	0/33
eftychiou *et al.*	21	31	11	73	53	17	27	10	61	47	0/9	0/43
Dogra *et al.*	120	55	9	295	73	250	91	9	591	109	0/834	0/15
Tomaiuolo *et al.*	574	754	298	1902	1350	311	436	162	1058	760	0/668	0/42
Söderström *et al.*	265	205	44	735	293	489	392	90	1370	572	0/373	0/29
Sakowicz *et al.*	160	98	14	418	126	91	47	3	229	53	0/274	0/19
slama *et al.*	16	82	2	114	86	104	79	17	287	113	0/717	0/28
Mehlig *et al.*	451	340	65	1242	470	586	464	79	1636	622	0/32	0/27
Mehlig *et al.*	160	118	16	438	150	328	242	54	898	350	0/328	0/28
Shaker *et al.*	44	12	4	100	20	50	10	0	110	10	0/481	0/08
Ezzat *et al.*	12	16	2	40	20	13	2	0	28	2	0/782	0/07
Al-Gazally *et al.*	16	8	6	40	20	19	6	5	44	16	0/007	0/26
Alkhiary *et al.*	14	16	1	44	18	5	15	0	25	15	0/007	0/37
Senol *et al.*	50	12	8	112	28	46	16	8	108	32	0/003	0/22
Hmimech *et al.*	38	52	10	128	72	95	76	11	266	98	0/408	0/26
Iqbal *et al.*	103	34	6	240	46	118	33	3	269	39	0/698	0/12
Zhang *et al.*	85	140	79	310	298	170	288	93	628	474	0/12	0/43
Hashad *et al.*	55	34	11	144	56	73	25	2	171	29	0/934	0/14
Garcia-Gonzalez *et al.*	18	42	19	78	80	25	41	35	91	111	0/07	0/54
Eissa *et al.*	42	25	8	109	41	36	32	7	104	46	0/976	0/30
Hou *et al.*	461	340	59	1262	458	607	424	66	1638	556	0/477	0/25
Zhang *et al.*	163	424	288	750	1000	220	469	267	909	1003	0/61	0/52
Long *et al.*	80	172	154	332	480	88	95	48	271	191	0/2	0/41
Raina *et al.*	358	38	4	754	46	391	9	0	791	9	0/819	0/01
Ameen *et al.*	56	33	13	145	59	40	25	12	105	49	0/027	0/31
Bouzidi *et al.*	131	121	58	383	237	123	78	6	324	90	0/122	0/21
Shivkar *et al.*	34	9	2	77	13	28	17	0	73	17	0/118	0/18
Golestani *et al.*	69	74	7	212	88	88	55	7	231	69	0/666	0/23
Sugijo *et al.*	25	4	1	54	6	22	8	0	52	8	0/399	0/13
Timizheva *et al.*	58	44	11	160	66	31	24	7	86	38	0/418	0/3
**Study author**	**MI cases**					**Healthy control**					**P-HWE**	**MAF**
	**AA**	**AC**	**CC**	**A**	**C**	**AA**	**AC**	**CC**	**A**	**C**		
Ranjith *et al.*	75	78	42	228	162	102	152	46	356	244	0/387	0/41
Angeline *et al.*	19	24	9	62	42	8	10	2	26	14	0/658	0/35
Dayakar *et al.*	80	56	16	216	88	139	27	1	305	29	0/8	0/09
eftychiou *et al.*	21	32	10	74	52	17	30	7	64	44	0/268	0/41
slama *et al.*	83	17	0	183	17	186	14	0	386	14	0/608	0/03
Soderstrom *et al.*	198	255	64	651	383	403	440	127	1246	694	0/688	0/36
Nasiri *et al.*	11	35	8	57	51	16	26	12	58	50	0/815	0/46
Alkhiary *et al.*	10	18	3	38	24	2	18	0	22	18	0/002	0/45
Senol *et al.*	39	17	14	95	45	36	18	16	90	50	0/002	0/35
Iqbal *et al.*	32	63	48	127	159	37	86	31	160	148	0/141	0/48
Garcia-Gonzalez *et al.*	56	22	1	134	24	77	21	3	175	27	0/304	0/13
Eissa *et al.*	36	31	8	103	47	37	28	10	102	48	0/218	0/32
Zhang *et al.*	567	256	52	1390	360	633	287	36	1553	359	0/626	0/18
Long *et al.*	294	100	12	688	124	152	73	6	377	85	0/425	0/18
Amani *et al.*	36	21	22	93	65	20	44	12	84	68	0/136	0/44
Shivkar *et al.*	4	28	13	36	54	6	35	4	47	43	0/001	0/47
Ameen *et al.*	34	48	20	116	88	32	29	16	93	61	0/061	0/39
Timizheva *et al.*	29	68	16	126	100	21	36	5	78	46	0/54	0/37

P-HWE, *P*-value for Hardy–Weinberg equilibrium; MAF, minor allele frequency of control group.

### 3.2 Meta-analysis of C677T SNP and MI risk

**Fig. 2:**
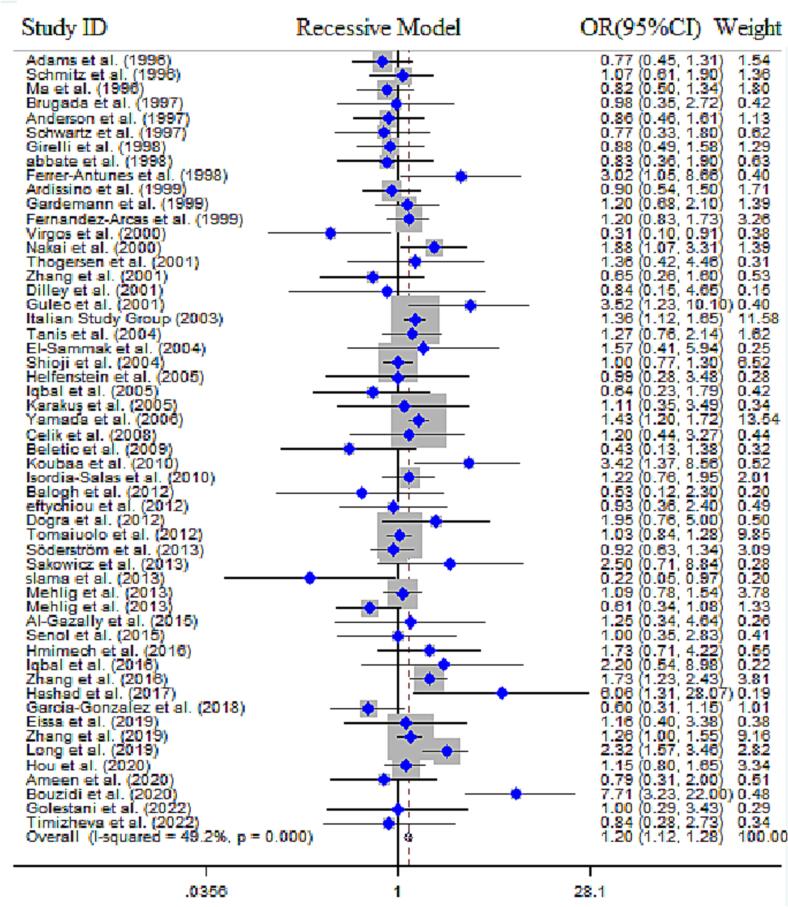

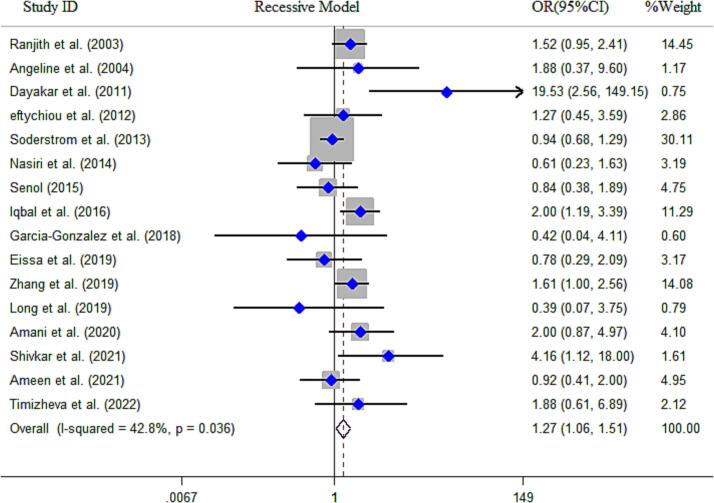
**A.** Pooled ORs and 95 % CI of individual studies and pooled data for the association between *MTHFR* gene C677T polymorphism and risk of MI in overall populations for recessive model. **B).** Pooled ORs and 95 % CI of individual studies and pooled data for the association between *MTHFR* gene A1298T polymorphism and risk of MI in overall populations for recessive model.

**Fig. 3:**
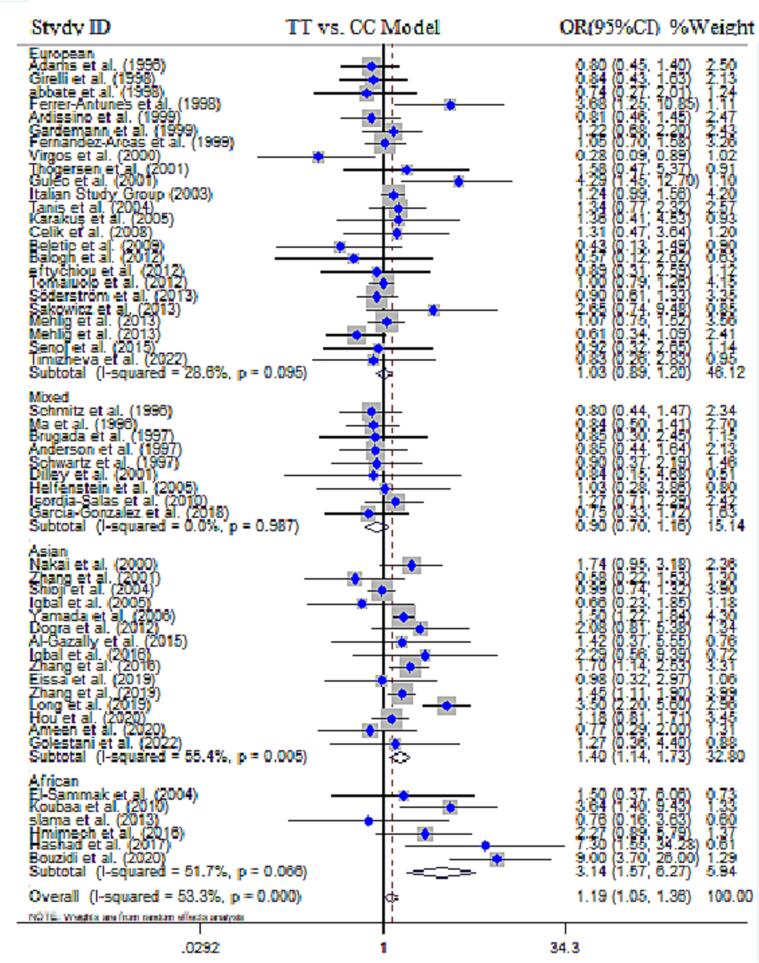
Pooled OR and 95 % CI of individual studies and pooled data for the association between *MTHFR* gene C677T polymorphism and risk of MI in different ethnicity based on subgroup analysis for TT *vs*. CC model.

**Fig. 4:**
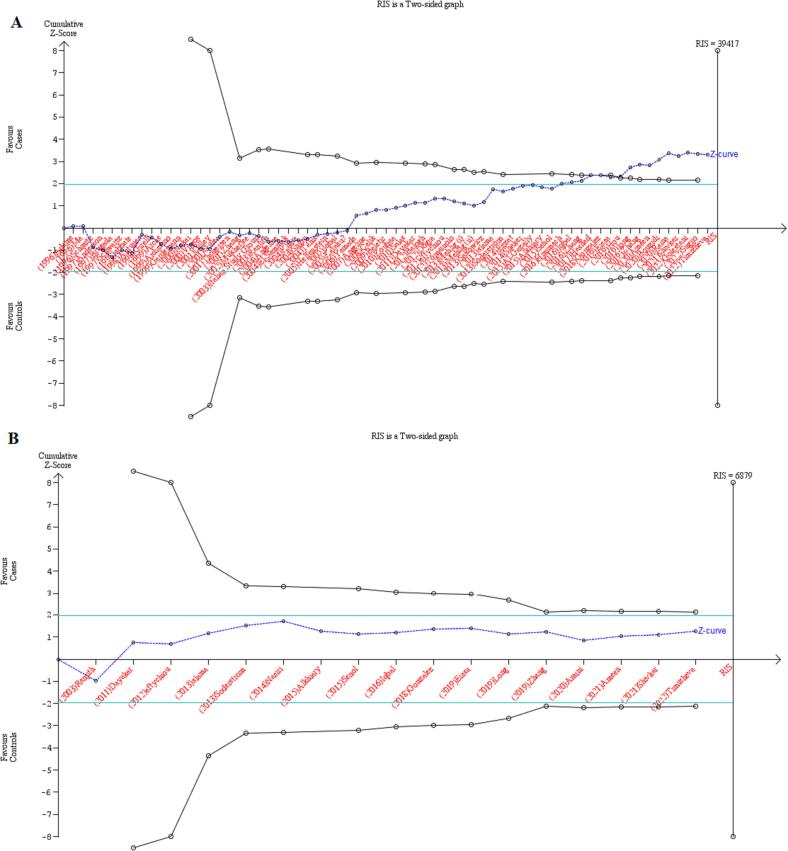
Trial sequence analysis (TSA) of all the included studies considering MTHFR gene polymorphism [C677T (A), A1298C (B)] based on dominant genetic model. Cumulative Z-curve (dashed blue line), conventional boundary (green horizontal lines), trial sequential monitoring boundaries (inward sloping black lines), required information sizes (black vertical line). (For interpretation of the references to colour in this figure legend, the reader is referred to the web version of this article.)

In current study, a total of 66 research papers were collected, which contained data regarding the association between the *MTHFR* gene C677T polymorphism and the risk of MI. The quantitative analysis encompassed a sample size of 16,860 cases and 20,403 controls. Out of the publications analyzed, 24 were performed in European countries, 23 were in Asian countries, 10 publications in Africans, and 9 studies in countries with mixed ethnicity (American, African, and Latin). The results of combined OR for overall population indicated a statistically significant association between *MTHFR* gene C677T SNP and MI risk across all five genotype models, including dominant model (OR = 1.16, 95 % CI = 1.06–1.28, *P* = 0.008, REM), recessive model (OR = 1.20, 95 % CI = 1.12–1.28, *P* < 0.001, REM), allelic model (OR = 1.13, 95 % CI = 1.06–1.21, *P* < 0.001, REM), TT *vs*. CC model (OR = 1.19, 95 % CI = 1.05–1.36, *P* < 0.001, FEM) and the CT *vs*. TT model (OR = 1.11, 95 % CI = 1.02–1.21, *P* = 0.01, FEM) (Fig. 2**)**. Alongside with these findings, the results of subgroup stratification based on ethnicity revealed that C677T SNP was significantly associated with MI risk in Asians, according to dominant model (OR = 1.27, 95 % CI = 1.07–1.51, *P* = 0.005, REM), recessive model (OR = 1.34, 95 % CI = 1.22–1.49, *P* < 0.001, FEM), allelic model (OR = 1.23, 95 % CI = 1.09–1.39, *P* = 0.001, REM), TT *vs*. CC model (OR = 1.40, 95 % CI = 1.14–1.73, *P* < 0.001, REM)], and the CT *vs*. TT model (OR = 1.19, 95 % CI = 1.03–1.37, *P* = 0.02, REM) in Africans by dominant model (OR = 1.73, 95 % CI = 1.11–2.70, *P* = 0.01, REM), recessive model (OR = 2.57, 95 % CI = 1.64–4.03, *P* < 0.001, REM), allelic model (OR = 1.59, 95 % CI = 1.17–2.15, *P* = 0.003, REM), and TT *vs*. CC model (OR = 3.14, 95 % CI = 1.57–6.27, *P* < 0.001, FEM) (Fig. 3). The cumulative Z-curve crossed the trial sequential monitoring boundaries and reached the RIS, thereby confirming the results of the conventional meta-analysis. Specifically, it affirmed the association between the C677T SNP and an increased risk of MI, indicating that further studies to establish this association are unnecessary (Fig. 4). However, no significant association was obtained in Europeans and peoples with mixed ethnic background (Table 3).

**Table 3:** Main results of pooled ORs in meta-analysis of *MTHFR* gene polymorphisms.

**Subgroup**		**Sample size**	**Test of association**		**Test of heterogeneity**		**Test of publication bias (Begg’s test)**		**Test of publication bias (Egger’s test)**	
	**Genetic model**	**Case/Control**	**OR**	**95% CI (*P*-value)**	**I^2^ (%)**	**P**	**Z**	**P**	**T**	**P**
**MTHFR C677T**										
**Overall**	Dominant	16860 / 20403	1.16	1.06 - 1.28 (0.008)	67.8	≤0.001	0.93	1	0.09	0.93
	Recessive	16860 / 20403	1.20	1.12 – 1.28 (<0.001)	49.2	≤0.001	0.76	0.67	-0.31	0.76
	Allelic	16860 / 20403	1.13	1.06 - 1.21 (<0.001)	65.8	≤0.001	0.47	0.45	-0.76	0.47
	TT vs. CC	16860 / 20403	1.19	1.05 – 1.36 (<0.001)	53.3	≤0.001	0.95	0.49	0.06	0.95
	CT *vs.* CC	16860 / 20403	1.11	1.02 – 1.21 (0.01)	54.4	≤0.001	0.91	0.62	-0.12	0.91
**Subgroup (Ethnicity)**										
**European**	Dominant	8030 / 8046	1.00	0.93 – 1.07 (0.94)	0	0.61	-1.88	0.06	-1.42	0.06
	Recessive	8030 / 8046	1.09	0.99 – 1.21 (0.07)	29.8	0.08	-1.52	0.12	-1.90	0.09
	Allelic	8030 / 8046	1.02	0.96 – 1.09 (0.48)	26.7	0.11	-0.68	0.49	-0.59	0.61
	TT vs. CC	8030 / 8046	1.03	0.89 – 1.20 (0.69)	28.6	0.07	-0.15	0.88	0.23	0.83
	CT *vs.* CC	8030 / 8046	0.98	0.91 – 1.05 (0.53)	0	0.83	1.35	0.17	1.24	0.26
**Mixed**	Dominant	1244 / 1696	0.93	0.80 - 1.10 (0.40)	0	0.53	0.94	0.34	1.64	0.17
	Recessive	1244 / 1696	0.92	0.73 - 1.15 (0.45)	0	0.87	0.68	0.38	1.59	0.06
	Allelic	1244 / 1696	0.94	0.84 - 1.06 (0.31)	0	0.85	0.98	0.32	2.59	0.08
	TT vs. CC	1244 / 1696	0.90	0.70 - 1.16 (0.41)	0	0.98	-0.68	0.49	-0.59	0.61
	CT *vs.* CC	1244 / 1696	0.95	0.79-1.16 (0.64)	19	0.27	0.45	0.65	0.54	0.61
**Asian**	Dominant	6551 / 9409	1.27	1.07 – 1.51 (0.005)	75	0.001	1.34	0.18	0.23	0.82
	Recessive	6551 / 9409	1.34	1.22 – 1.49 (<0.001)	42.5	0.04	1.46	0.14	1.35	0.22
	Allelic	6551 / 9409	1.23	1.09 - 1.39 (0.001)	68.4	0.008	-1.36	0.17	-1.69	0.22
	TT vs. CC	6551 / 9409	1.40	1.14 – 1.73 (<0.001)	55.4	0.005	-0.25	0.80	-0.68	0.52
	CT *vs.* CC	6551 / 9409	1.19	1.03 – 1.37 (0.02)	58.3	0.06	1.47	0.14	2.01	0.13
**African**	Dominant	1035 / 1252	1.73	1.11 – 2.70 (0.01)	78.8	≤0.001	2.47	0.09	3.38	0.01
	Recessive	1035 / 1252	2.57	1.64 – 4.03 (<0.001)	72.9	0.001	-0.42	0.67	-0.48	0.64
	Allelic	1035 / 1252	1.59	1.17 – 2.15 (0.003)	72.6	≤0.001	0.46	0.64	-0.04	0.96
	TT vs. CC	1035 / 1252	3.14	1.57 – 6.27 (<0.001)	51.7	0.06	-0.10	0.92	0.49	0.63
	CT *vs.* CC	1035 / 1252	1.54	0.96 – 2.46 (0.07)	79.3	≤0.001	0.68	0.49	0.48	0.67
**MTHFR A1298C**										
**Overall**	Dominant	3162 / 3632	1.13	0.91 – 1.42 (0.27)	66.8	≤0.001	-0.21	0.83	0.2	0.82
	Recessive	3162 / 3632	1.27	1.06 – 1.51 (0.008)	42.8	0.03	1.04	0.29	0.62	0.55
	Allelic	3162 / 3632	1.18	1.01 – 1.39 (0.03)	66.8	≤0.001	-1.73	0.08	-0.69	0.51
	CC vs. AA	3162 / 3632	1.22	1.01 – 1.47 (0.04)	17.1	0.25	1.34	0.17	1.49	0.14
	AC *vs.* AA	3162 / 3632	1.08	0.85– 1.37 (0.53)	69.3	≤0.001	1.39	0.16	1.54	0.13
**Subgroup (Ethnicity)**										
**European**	Dominant	763 / 1156	1.13	0.93 – 1.37 (0.23)	0	0.67	1.24	0.21	0.74	0.48
	Recessive	763 / 1156	0.98	0.74 - 1.30 (0.89)	0	0.67	1.99	0.13	0.65	0.37
	Allelic	763 / 1156	1.06	0.93 – 1.22(0.39)	0	0.59	1.37	0.16	1.58	0.13
	CC vs. AA	763 / 1156	0.98	0.73– 1.33 (0.90)	0	0.88	1.96	0.73	1.65	0.61
	AC *vs.* AA	763 / 1156	1.14	0.93 – 1.40 (0.21)	0	0.78	0.19	0.85	-0.53	0.62
**Asian**	Dominant	1994 / 1855	1.18	0.81 – 1.71 (0.38)	77	0.002	0.71	0.47	0.87	0.39
	Recessive	1994 / 1855	1.53	1.18 – 1.99 (0.002)	50.2	0.03	1.70	0.09	1.78	0.08
	Allelic	1994 / 1855	1.25	0.95 – 1.64 (0.10)	78.5	≤0.001	-1.23	0.47	-1.88	0.11
	CC vs. AA	1994 / 1855	1.49	1.12- 1.98 (0.006)	30.1	0.16	0.83	0.40	0.62	0.54
	AC *vs.* AA	1994 / 1855	1.09	0.75 – 1.58 (0.66)	76.8	0.004	-1.23	0.47	-0.95	0.38
**African**	Dominant	326 / 520	0.96	0.33– 2.80 (0.94)	78.7	0.009	1.57	0.11	1.09	0.29
	Recessive	326 / 520	1.52	0.95 – 2.41 (0.07)	0	0.58	1.24	0.21	0.39	0.70
	Allelic	326 / 520	1.24	0.69 – 2.24 (0.47)	67.2	0.04	0.56	0.57	1.87	0.13
	CC vs. AA	326 / 520	1.24	0.74 – 2.08 (0.40)	0	0.76	0.52	0.60	0.94	0.52
	AC *vs.* AA	326 / 520	0.85	0.26 – 2.80 (0.79)	84.6	0.002	0.63	0.53	0.31	0.76

### 3.3 Meta-analysis of A1298C SNP and MI risk

In total, the final analysis included 18 studies comprising 3,162 cases and 3,632 controls to examine the potential association between the *MTHFR* gene A1298C polymorphism and the risk of MI. Among the included studies, four publications were conducted in European countries, ten publications were in Asian countries, three publications in Africans, and only one study in a country with mixed ethnicity (Mexico).

Our findings indicated significant association between A1298C SNP and the risk of MI under recessive model (OR = 1.27, 95 % CI = 1.06–1.51, *P* = 0.008, FEM), allelic model (OR = 1.18, 95 % CI = 1.01–1.39, *P* = 0.03, FEM), and CC *vs*. AA model (OR = 1.22, 95 % CI = 1.01–1.47, *P* = 0.04, FEM). The results of TSA revealed that the cumulative Z-curve did not intersect any of trial sequential monitoring boundary and the RIS, suggesting more trials are required to reach a robust conclusion (Fig. 4). Furthermore, when conducting subgroup analysis based on ethnicity, no significant association was observed among individuals of African and European. However, two models, including recessive model (OR = 1.53, 95 % CI = 1.18–1.99, *P* = 0.002, FEM) and CC *vs*. AA model (OR = 1.49, 95 % CI = 1.12–1.98, *P* = 0.006, FEM) had significant associations with MI risk in Asians. Due to the limited availability of data from only one study involving individuals with mixed ethnicity, we excluded it from the subgroup analysis.

### 3.4 Meta-regression analyses

**Fig. 5:**
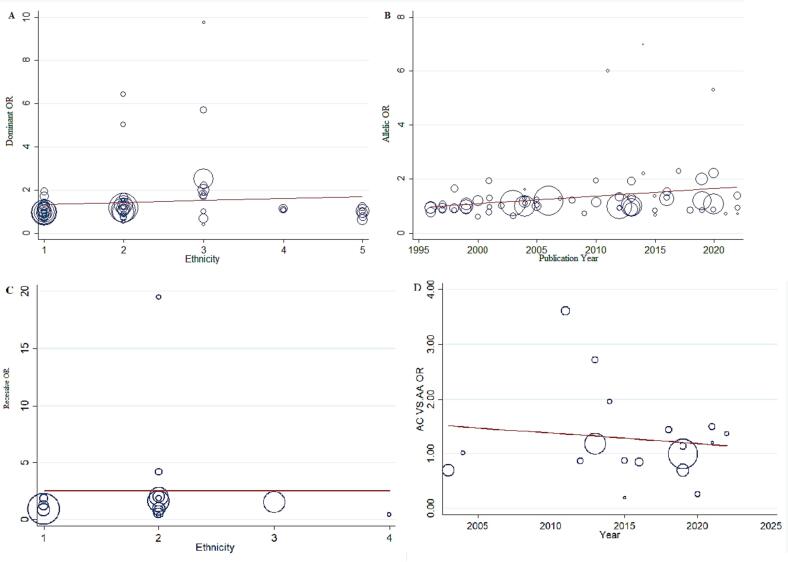
Meta-regression plots of the association between *MTHFR* gene polymorphisms and risk of MI based on A: ethnicity (C677T), B: publication year (C677T), C: ethnicity (A1298C), D: publication year (A1298C).

Meta-regression analysis was conducted for *MTHFR* gene C677T and A1298C polymorphisms to find potential sources of heterogeneity. However, the results indicated that none of the potential factors contributed to the existing heterogeneity (all *P*-values more than 0.05; Table 4**,** Fig. 5**)**.

**Table 4:** Meta-regression analyses of potential source of heterogeneity.

	Heterogeneity Factor	Coefficient	SE	T-test	P-value	95 % CI	
						UL	LL
MTHFR C677T							
Publication Year	Dominant	0.045	0.025	1.81	0.07	−0.004	0.096
	Recessive	0.019	0.016	1.13	0.26	−0.015	0.053
	Allelic	0.028	0.016	1.68	0.09	−0.005	0.061
	TT *vs*. CC	0.029	0.021	1.35	0.18	−0.014	0.072
	CT *vs.* CC	0.043	0.024	1.75	0.08	−0.006	0.092
Ethnicity	Dominant	0.076	0.138	0.55	0. 58	−0.202	0.354
	Recessive	−0.019	0.088	−0.22	0.82	−0.198	0.159
	Allelic	0.021	0.091	−0.24	0.81	−0.160	0.204
	TT *vs*. CC	−0.018	0.114	−0.16	0.87	−0.249	0.211
	CT *vs.* CC	0.079	0.134	0.59	0.55	−0.189	0.348
MTHFR A1298C							
Publication Year	Dominant	−0.029	0.044	−0.65	0.52	−0.124	0.066
	Recessive	−0.141	0.208	−0.68	0.50	−0.588	0.304
	Allelic	−0.029	0.037	−0.78	0.44	−0.109	0.050
	CC *vs*. AA	−0.223	0.302	−0.74	0.47	−0.872	0.426
	AC *vs.* AA	0.019	0.039	−0.51	0.61	−0.103	0.063
Ethnicity	Dominant	0.089	0.304	0.30	0.77	−0.554	0.734
	Recessive	0.017	1.589	0.01	0.99	−3.392	3.426
	Allelic	0.096	0.262	0.37	0.71	−0.459	0.652
	CC *vs*. AA	0.181	2.307	0.08	0.93	−4.767	5.130
	AC *vs.* AA	0.122	0.264	0.46	0.65	−0.438	0.683

### 3.5 Assessment of publication bias and heterogeneity

**Fig. 6:**
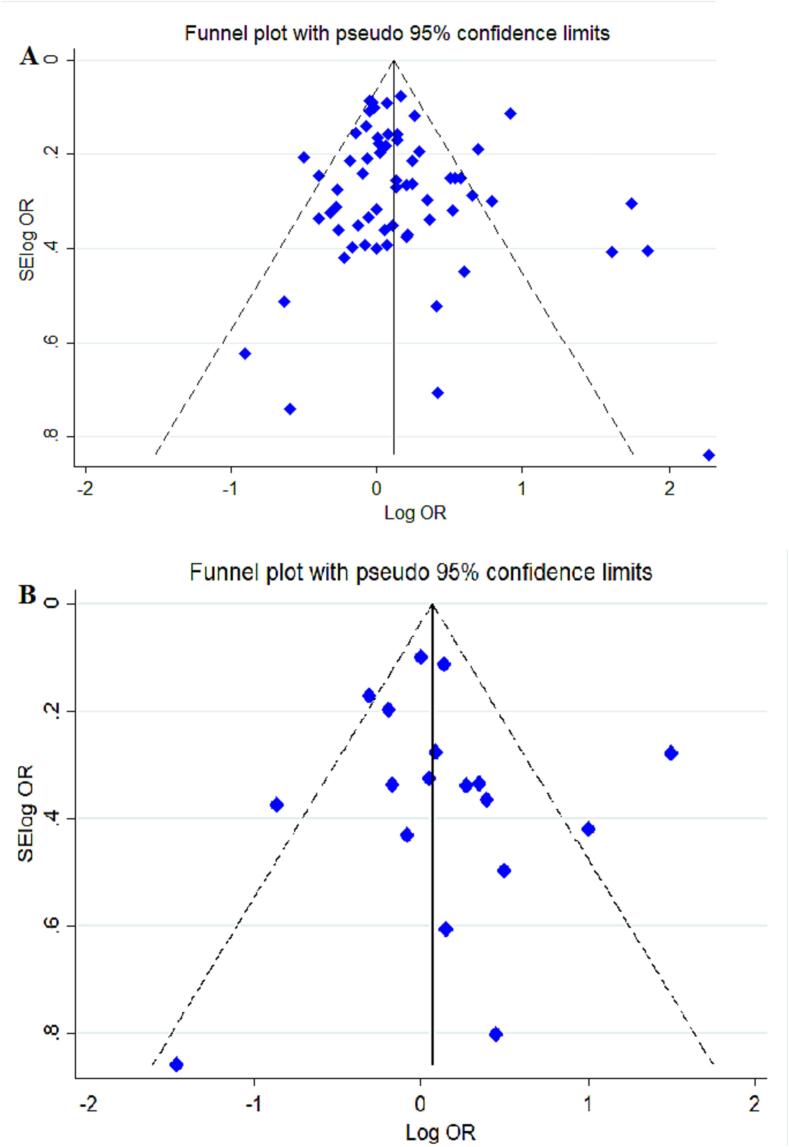
Begg’s funnel plot for publication bias test for the association between *MTHFR* gene polymorphisms and risk of MI in the dominant model A: C677T polymorphism, B: A1298C polymorphism. Each point represents a separate study for the indicated association.

**Fig. 7:**
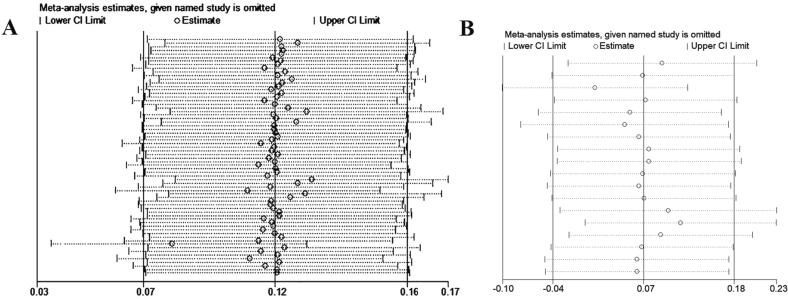
Sensitivity analysis to investigate the *MTHFR* gene polymorphisms contributing to MI risk (A, C677T polymorphism; B, A1298C polymorphism).

The study employed Begg's funnel plot visualization and Egger's weighted regression test to assess publication bias. No significant publication bias was observed based on the findings, as illustrated in Fig. 6. Additionally, to evaluate the impact of each individual study on the overall estimation, a sensitivity analysis was conducted by systematically excluding one study at a time, thereby examining the potential influence of each study on the final result. The results showed that none of the publications had a significant effect on the pooled ORs in the dominant model for C677T and A1298C SNPs, as demonstrated in Fig. 7.

## 4 Discussion

The metabolism of homocysteine involves an enzyme called MTHFR, which plays a key role in converting 5,10-methylenetetrahydrofolate to 5-methyltetrahydrofolate. This enzymatic reaction is critical for the production of methionine from homocysteine [97]. Two common SNPs in the MTHFR gene, C667T/*rs1801133* and A1298C/*rs1801131*, have been associated with hyperhomocysteinemia and an increased risk of developing cardiovascular disease (CVD) [98]. The *MTHFR* gene polymorphisms are particularly important in the context of MI susceptibility due to their involvement in the one-carbon metabolism pathway, which has significant mechanistic implications. These genetic variations can lead to reduced MTHFR enzymatic activity, resulting in lower levels of active folate and impaired homocysteine metabolism. Elevated homocysteine levels, which are a consequence of specific *MTHFR* polymorphisms, have been linked to endothelial dysfunction, oxidative stress, inflammation, and an increased propensity for blood clot formation. These factors collectively contribute to the development of atherosclerosis, a precursor to MI [99]. Additionally, inadequate folate availability can compromise DNA methylation processes, which can affect the expression of genes involved in cardiovascular health and lipid metabolism. The mechanistic implication is that individuals with specific *MTHFR* gene polymorphisms may have an increased risk of MI due to these interconnected factors [100]. Understanding the underlying mechanisms of this susceptibility is crucial, as it can inform interventions and personalized strategies targeting homocysteine metabolism and DNA methylation, potentially reducing the risk of MI for individuals with a genetic predisposition.

Performing a meta-analysis study on the relationship between *MTHFR* gene polymorphisms and susceptibility to MI is of great importance due to its numerous advantages. This approach aims to consolidate findings from multiple individual studies, effectively pooling evidence to provide a comprehensive and accurate assessment of this genetic association. By gathering data from various sources, meta-analysis enhances statistical power, enabling the detection of subtle associations that may not be apparent in individual investigations. Additionally, it facilitates a thorough exploration of sources of heterogeneity, shedding light on the reasons for variations in findings among studies. By resolving conflicts and distinguishing genuine associations from spurious ones, meta-analysis produces robust effect size estimates and contributes to well-informed clinical practices. It also addresses publication bias by assessing selective publication of significant results. By illuminating these aspects, meta-analysis not only advances scientific knowledge but also has the potential to influence clinical strategies, risk assessment, and personalized treatment approaches for individuals with specific *MTHFR* gene polymorphisms and those at risk of MI. Therefore, we conducted an up-to-date meta-analysis of available data on *MTHFR* gene C667T and A1298C SNPs in relation to predisposition to MI. Our analysis revealed a significant association between C677T and A1298C SNPs and the risk of MI in the pooled analysis.

The initial meta-analysis investigating the association between *MTHFR* gene SNPs and the risk of myocardial infarction (MI) was conducted by Xuan et al. in 2011 [101]. They examined the *MTHFR* gene C677T SNP by including 30 studies, which encompassed a total of 10,522 controls and 8,140 MI cases. The study findings suggested that the TT *vs*. CT model (OR = 1.18) of the *MTHFR* gene C677T SNP was associated with an increased risk of MI. Subsequently, in 2016, Alizadeh et al. [102] performed a meta-analysis on 47 studies (encompassing 15,865 controls and 12,637 cases) for the *MTHFR* gene C677T SNP and 7 studies (including 1,765 controls and 1,133 cases) for the A1298C SNP in relation to MI. Their pooled analysis indicated that none of the five genetic models for both C677T and A1298C polymorphisms showed significant associations with MI risk. In our analysis, we incorporated 66 studies (comprising 16,860 cases and 20,403 controls) for the *MTHFR* gene C677T polymorphism and 18 studies (including 3,162 cases and 3,632 controls) for the A1298C polymorphism concerning the risk of MI. Our analysis revealed significant associations between four models of the *MTHFR* gene C677T polymorphism and an increased risk of MI. These models include the dominant model (OR = 1.16), recessive model (OR = 1.20), allelic model (OR = 1.13), TT *vs*. CC model (OR = 1.19), and CT *vs*. CC model (OR = 1.11). Additionally, our analysis demonstrated a significant association between the A1298C SNP and the risk of MI under the recessive model (OR = 1.27), allelic model (OR = 1.18), and TT *vs*. CC genotypic model (OR = 1.22) in the overall analysis.

The subgroup analysis conducted in the study by Xuan et al. [101] in 2011 revealed that the TT *vs*. CT model of the *MTHFR* gene C677T SNP increased the risk of MI in Caucasians (OR = 1.13) and young/middle-aged Caucasians (<50 years) (OR = 1.27). Additionally, Alizadeh et al. [102] indicated that the T allele of the C677T SNP was associated with a higher risk of MI (OR = 1.63) in African cases. However, in North American populations, the CT genotype was associated with a lower risk of MI (OR = 0.81). Moreover, the subgroup analysis based on ethnicity and gender found no association between the A1298C SNP and the risk of MI. In contrast, our analysis demonstrated significant associations between the C677T SNP and MI risk in Asians, according to the dominant model (OR = 1.27), recessive model (OR = 1.34), T allele (OR = 1.23), and TT genotype (OR = 1.40), as well as in Africans based on the dominant model (OR = 1.73), recessive model (OR = 2.57), T allele (OR = 1.59), TT genotype (OR = 3.14), and CT genotype (OR = 1.54). Furthermore, our analysis indicated that the recessive model (OR = 1.53) and CC genotype (OR = 1.49) of the A1298C SNP increased the risk of MI in the Asian population. These conflicting results may be attributed to relatively smaller sample sizes in previous meta-analyses, and it appears that our analysis provides more robust statistical power to establish a more valid association. Additionally, our meta-regression analyses revealed that the ethnicity of the subjects and publication year were not potential sources of heterogeneity in the pooled analysis of both C677T and A1298C polymorphisms.

The TSA conducted in this study provided valuable insights into the available data regarding the association between the *MTHFR* gene C677T SNP and MI. The results of the TSA analysis demonstrated that the existing body of data was substantial and robust enough to establish a valid and conclusive association between the *MTHFR* gene C677T SNP and susceptibility to MI. This indicates that the available evidence was not only consistent but also sufficient to support a strong and reliable relationship between the C677T SNP and the risk of MI. Conversely, when examining the *MTHFR* gene A1298C SNP, the TSA analysis revealed a different perspective. The analysis indicated that the studies available at the time were insufficient to establish a conclusive association between the A1298C SNP and MI. In other words, the existing data did not reach a level of adequacy to provide a definitive and reliable association between this specific SNP and the risk of MI. These findings highlight the necessity for further research and studies dedicated to the investigation of the *MTHFR* gene A1298C SNP to obtain a more comprehensive and conclusive understanding of its relationship with MI. It is evident that additional investigations are essential to gather enough data and evidence to support a valid association between the A1298C SNP and the risk of MI.

This meta-analysis provides valuable insights into the association between *MTHFR* gene polymorphisms and MI susceptibility, but it is important to acknowledge several limitations that warrant consideration and suggest future research directions. Firstly, it is worth noting that six out of the 68 studies included in this analysis had control groups with deviations from HWE. This discrepancy in control group genotypes could potentially introduce bias into the original studies and may contribute to heterogeneity in the overall analysis. Although our analysis did not identify significant heterogeneity, addressing the issue of HWE deviations in control groups is crucial to enhance the robustness of future studies in this field. Secondly, MI is recognized as a polygenic disease, meaning that multiple genes and their interactions contribute to MI susceptibility. Exploring gene-to-gene interactions, haplotype associations, and gene-environment interactions can provide a more comprehensive understanding of MI predisposition. Unfortunately, due to the limited availability of well-structured data in the included studies, we were unable to conduct such analyses in this meta-analysis. Future investigations should aim to explore these complex genetic interactions to unravel the full spectrum of genetic contributors to MI risk. Lastly, it is important to highlight that there was a limited number of studies specifically related to the *MTHFR* gene A1298C polymorphism, resulting in a relatively small data pool for this specific polymorphism. To overcome this limitation and obtain a more comprehensive understanding of the involvement of this functional SNP in MI risk, further research is strongly recommended. These future studies should encompass diverse ethnic populations to ensure broader applicability of the findings and better representation of the genetic diversity in different communities. Taken together, while this meta-analysis advances our knowledge of the role of the *MTHFR* gene in MI susceptibility, it is important to address these limitations in future research endeavors. This will help refine our understanding of the genetic associations with MI and provide more personalized insights into risk assessment and clinical strategies for individuals with specific *MTHFR* gene polymorphisms.

## 5 Conclusion

In conclusion, this comprehensive and up-to-date meta-analysis has shed light on the significant association between *MTHFR* gene SNPs and susceptibility to MI. The analysis encompassed various genetic models for *MTHFR* gene C677T polymorphism, including the dominant, allelic, recessive, CT *vs*. CC, and TT *vs*. CC models, all of which exhibited a substantial link with an increased risk of MI. Furthermore, our analysis revealed a noteworthy association between the A1298C SNP and MI risk under three different genotype models when considering the entire dataset. Interestingly, when we delved into specific ethnic populations, we observed variations in these genetic associations. For the C677T SNP, it was significantly linked to MI risk in both Asian and African populations. On the other hand, the A1298C SNP demonstrated a propensity to elevate MI risk in the Asian population. These findings highlight the importance of considering genetic diversity and ethnicity when exploring the relationship between *MTHFR* gene polymorphisms and MI susceptibility. However, despite the valuable insights gained from this meta-analysis, there is still a need for further research, particularly regarding the *MTHFR* gene A1298C SNP. To obtain a thorough and unequivocal understanding of the *MTHFR* gene involvement in association with MI, additional studies are warranted. These future investigations should encompass diverse populations and ethnicities to provide a more comprehensive and universally applicable insight into the intricate genetic factors contributing to MI risk.

## Declaration of competing interest

The authors declare that they have no known competing financial interests or personal relationships that could have appeared to influence the work reported in this paper.
