# Supplementary material for: MTHFR gene polymorphisms and susceptibility to myocardial infarction: Evidence from meta-analysis and trial sequential analysis
Source: Int J Cardiol Heart Vasc. 2023 Nov 22;49:101293. doi: 10.1016/j.ijcha.2023.101293 (PMC10687297; doi:10.1016/j.ijcha.2023.101293)
Supplement: Supplementary data 1 [file mmc1.docx]

**Search strategy**

**PubMed**

Search: ((((“Methylenetetrahydrofolate Reductase” [Title/Abstract]) OR (“MTHFR” [Title/Abstract])) OR (C667T [Title/Abstract])) OR (A1298C [Title/Abstract])))) AND ((((((“SNP” [Title/Abstract]) OR (“single nucleotide polymorphism” [Title/Abstract])) OR (“polymorphisms” [Title/Abstract])) OR (“variation” [Title/Abstract])) OR (“mutation” [Title/Abstract]))AND ((((“Myocardial Infarction” [Title/Abstract]) OR (“Acute coronary syndrome” [Title/Abstract])) (“ischemic heart disease” [Title/Abstract])) OR (“coronary artery disease” [Title/Abstract]))

Study number=654

**Scopus**

#3 AND #2 AND #1

#3 (( TITLE-ABS-KEY (Methylenetetrahydrofolate Reductase)  OR  TITLE-ABS-KEY (MTHFR)  OR  TITLE-ABS-KEY (C667T)  OR  TITLE-ABS-KEY (A1298C)

AND

#2 (( TITLE-ABS-KEY (SNP) OR TITLE-ABS-KEY (single nucleotide polymorphism) OR TITLE-ABS-KEY (polymorphisms) OR TITLE-ABS-KEY (variation)) OR TITLE-ABS-KEY (mutation)

AND

#1 ( (TITLE-ABS-KEY (Myocardial Infarction) OR  TITLE-ABS-KEY (Acute coronary syndrome)  OR TITLE-ABS-KEY (ischemic heart disease) OR TITLE-ABS-KEY (coronary artery disease)

Study number=1216

**Web of Science**

#1 AND #2 AND #3

#1TOPIC:  (Myocardial Infarction)  OR  TOPIC:  (Acute coronary syndrome) OR  TOPIC:  (ischemic heart disease)OR  TOPIC:  (coronary artery disease)  AND

#2TOPIC: (SNP)  OR  TOPIC:  (single nucleotide polymorphism)  OR TOPIC:  (polymorphisms)  OR  TOPIC:  (variation) OR  TOPIC:  (mutation) AND

#3TOPIC: (Methylenetetrahydrofolate Reductase) OR  TOPIC:  (MTHFR)  OR

TOPIC:  (C667T)  OR  TOPIC:  (A1298C)

Study number=350
